# Effects of Mental Load and Fatigue on Steady-State Evoked Potential Based Brain Computer Interface Tasks: A Comparison of Periodic Flickering and Motion-Reversal Based Visual Attention

**DOI:** 10.1371/journal.pone.0163426

**Published:** 2016-09-22

**Authors:** Jun Xie, Guanghua Xu, Jing Wang, Min Li, Chengcheng Han, Yaguang Jia

**Affiliations:** 1 School of Mechanical Engineering, Xi’an Jiaotong University, Xi’an, Shaanxi, People’s Republic of China; 2 State Key Laboratory for Manufacturing Systems Engineering, Xi’an Jiaotong University, Xi’an, Shaanxi, People’s Republic of China; 3 School of Software Engineering, Xi’an Jiaotong University, Xi’an, Shaanxi, People’s Republic of China; Centre de neuroscience cognitive, FRANCE

## Abstract

Steady-state visual evoked potentials (SSVEP) based paradigm is a conventional BCI method with the advantages of high information transfer rate, high tolerance to artifacts and the robust performance across users. But the occurrence of mental load and fatigue when users stare at flickering stimuli is a critical problem in implementation of SSVEP-based BCIs. Based on electroencephalography (EEG) power indices *α*, *θ*, *θ* + *α*, ratio index *θ*/*α* and response properties of amplitude and SNR, this study quantitatively evaluated the mental load and fatigue in both of conventional flickering and the novel motion-reversal visual attention tasks. Results over nine subjects revealed significant mental load alleviation in motion-reversal task rather than flickering task. The interaction between factors of “stimulation type” and “fatigue level” also illustrated the motion-reversal stimulation as a superior anti-fatigue solution for long-term BCI operation. Taken together, our work provided an objective method favorable for the design of more practically applicable steady-state evoked potential based BCIs.

## Introduction

Brain-computer interfaces (BCIs) traditionally harness brain signals to control devices. By bypassing usual channels of muscle and peripheral nervous system, they could potentially be helpful for disabled individuals to control external devices or interact with the environment [1]. For the operations of BCI systems, high intrinsic cognitive or mental resources are usually needed to be involved in, which would potentially lead to high mental load and fatigue. Mental load has been extensively documented and can be defined generally as a measure of the amount of mental resources engaged in a task [2]. It is considered to be a measure of task difficulty. The mental fatigue, which is induced by cognitive load, is associated with tiredness and reduced arousal level and a decrease in task performance [3]. BCI users usually have a high cognitive load due to increased attentional demands from distractions, emotions, and interactions with other people. And when people become fatigued, they find it difficult to maintain vigilance level and to divide their attention between visual stimuli [4]. So the mental load and fatigue should be considered when designing BCI systems that require high visual and cognitive demands.

As one of the Electroencephalography (EEG) based BCI systems [5, 6], steady-state visual evoked potential (SSVEP) -based BCI paradigm, with the advantages of high information transfer rate, high tolerance to artifacts and the robust performance across users, has been widely used in application of BCIs. Most conventional SSVEP-based BCIs are based on flicker or contrast-change related paradigms, which enable users to perform the visual attention task [7]. The occurrence of mental load and fatigue when users stare at flickering stimuli is one of the critical problems in SSVEP-based BCI design. Previous studies have demonstrated that the amplitude and signal-to-noise ratio (SNR) of the elicited SSVEP are significantly affected by user’s fatigue. Once mental fatigue developed, SSVEP amplitude and SNR decreased [8]. Our preliminary study [9] presented a motion-reversal based steady-state motion visual evoked potential (SSMVEP) BCI, which may have the potential of being a user-friendly stimulus to alleviate mental load and visual fatigue caused by uncomfortable light twinkling and contrast changes [10, 11].

On this basis, the purpose of this study is to evaluate the effects of mental load and fatigue between prolonged operation of a traditional flickering visual attention task and the motion-reversal visual attention task. Considering increased mental load is most associated with a decrease of the EEG power in the α band (~8–13 Hz) and an increase of the EEG power in the θ band (~4–7 Hz) [12, 13], and increased fatigue level is often related to the global increases of EEG power in the α and θ bands [13], two objective analyzes are implemented. One analysis mainly measures the mental load effect between flickering SSVEP paradigm and motion-reversal SSMVEP paradigm based on EEG spectral power, in particular the *α* and *θ* band power and the ratio index *θ*/*α* that were acquired with the BCI tasks. And another analysis considers the mental-load-induced fatigue that occurred in four different fatigue levels in the visual attention task. The analysis is based on EEG spectral powers of *α*, *θ* and *θ* + *α*, and steady-state evoked potential properties of amplitude and SNR.

## Materials and Methods

### 1 Subjects and Recordings

Nine subjects (among which three female subjects), aged between 23 and 29 years old, are graduate students from Xi’an Jiaotong University (Shaanxi, China). All had normal or corrected-to-normal eyesight and had never experienced conventional flickering SSVEP- or motion-reversal SSMVEP-based BCIs before. They had no history of psychiatric or neurological disorders. And no light or motion perception disturbances or impairments were reported. All subjects were given informed written consent in compliance with the guidelines approved by the institutional review board of Xi’an Jiaotong University before the experiments.

In this study, EEG signals were recorded from the occipital (Oz) site using a single active gold-cup electrode with a ground electrode placed on the forehead (Fz). A reference electrode was attached to a unilateral earlobe. EEG signals were recorded by a g.USBamp (g.tec Inc., Austria) system at a sampling rate of 1200 Hz. An online band-pass filter from 2 to 100 Hz and the notch filter between 48 and 52 Hz were imposed to remove power line interference and to minimize low frequency components. All electrode impedances were kept below 5K ohms during experiments.

### 2 Stimulation Designs

Periodic flickering and motion-reversal stimulation paradigms were introduced as two separate experimental tasks. For each task, four stimulators were simultaneously presented to subjects through a gamma-corrected 22” Dell LCD monitor at a resolution of 1024 × 768 pixels and refresh rate of 60 Hz. Subjects were seated 70 cm from the computer screen with the center at eye level. Four stimulators were uniformly arranged in a quadrate with the eccentricity of 7.2° visual angle from the center of the monitor to that of each stimulator and each stimulator was created using a circular object of 4.8° diameter, in accordance with previous studies showing that a stimulus size beyond 3.8° would saturate VEP responses [14]. For the periodic flickering paradigm, each stimulator flickered between white and black and the brightness changes from light to dark and back to light were temporally sinusoidally modulated at a unique, constant frequency. The periodic motion-reversal paradigm utilized Newton’s rings as the motion-reversal stimulators [9]. Each stimulator was composed of five concentric and alternate black and white rings (Michelson contrast of 98.8%). The phase of the Newton’s ring was temporally sinusoidally modulated so as to produce the inward contraction and outward dilation motion-reversal procedure, which was scheduled according to our earlier study [9]. The mutually irrational reversal frequencies of 8, 12, 13.33 and 15 Hz were assigned to left, right, top and down stimulators, respectively. In the whole experiment, spatially homogeneous grey background was displayed in pauses and around the stimulators. Presentation of the stimulation was controlled by Psychophysics Toolbox (http://psychtoolbox.org/) [15, 16].

### 3 Experimental Setups

Subjects were requested to sit on an armchair in a dimly lit room with no electromagnetic shielding. For each subject, two experimental tasks were carried out for flickering SSVEP BCI and motion-reversal SSMVEP BCI, respectively. The four stimulators were simultaneously presented and subjects were instructed to binocularly maintain attention only on the target (i.e., down position) stimulator with stimulation frequency of 15 Hz throughout the experiments. Each task contained 4–8 runs and each run consisted of 20 epochs. Each epoch lasted 5 s and the inter-stimulus interval (ISI) was fixed to 5 s. So SSVEPs and SSMVEPs were recorded 195 s for each single run. The order of the flickering SSVEP-BCI and motion-reversal SSMVEP-BCI runs for each subject was alternated to avoid adaptation of long-term stimulation that may potentially affect assessment of mental load and fatigue effects. Subjects were not allowed to blink eyes or move their bodies during each run and they were asked to fixate on the center of screen during the ISI periods. Several minutes were reserved between runs as long as subjects wished. The whole experiment of each subject usually lasted about 25–40 min, depending on the inter-run relaxation governed by subjects. Therefore, horizontal or vertical electrooculography signals were not recorded and epochs contaminated by few artifacts were also not excluded.

### 4. Offline Target Recognition

CCA is a nonparametric multivariable method [17] to reveal the underlying correlation between two sets of multidimensional variables. It finds a pair of linear transforms for the two sets such that the transformed two sets have maximum correlation. Compared with traditional SSVEP recognition methods, it combines two steps of feature selection and frequency recognition, and therefore has been widely used in SSVEP-based BCIs [18,19].

Suppose that there are *K* stimulus frequencies *f*_1_, …, *f*_*K*_ in the BCI, for the calculation of correlation coefficients between stimulus frequency *f*_*i*_(*i* = 1, …, *K*) and EEG responses, two sets of signals are introduced into CCA. One set is the EEG signals *X* recorded from *C* different channels with time window of *S* sample points, and the other set is the stimulus signals *Y*_*i*_, which are composed of sinusoids and cosinusoids pairs at the same frequency of the stimulus and its harmonics. Stimulus signals *Y*_*i*_ are constructed as

$$Y_{i}=\left(\begin{array}{c} \text{cos}\left(2\pi \cdot f_{i}\cdot t\right)\\ \text{sin}\left(2\pi \cdot f_{i}\cdot t\right)\\ \vdots \\ \text{cos}\left(2\pi \cdot Hf_{i}\cdot t\right)\\ \text{sin}\left(2\pi \cdot Hf_{i}\cdot t\right) \end{array}\right),t=\frac{1}{Fs},\ldots ,\frac{S}{Fs}$$ (1)

where *Fs* is the sampling rate, and *H* is the number of harmonics.

Given the multidimensional variables of *X* and *Y*_*i*_, and their linear transformations *x* = *X*^*T*^*W*_*x*_ and $y_{i}\;=\;Y_{i}^{T}W_{y_{i}}$, CCA can find the weight vectors *W*_*x*_ and $W_{y_{i}}$ to maximize the canonical correlation of *x* and *y*_*i*_ (*i* = 1, …, *K*) through solving the following problem

$$\underset{W_{x},\;W_{y_{i}}}{\text{max}}\rho \left(x,y_{i}\right)=\frac{E\left(x^{T}y_{i}\right)}{\sqrt{E\left(x^{T}x\right)E\left(y_{i}^{T}y_{i}\right)}}=\frac{E\left(W_{x}^{T}XY_{i}^{T}W_{y_{i}}\right)}{\sqrt{E\left(W_{x}^{T}XX^{T}W_{x}\right)E\left(W_{y_{i}}^{T}Y_{i}Y_{i}^{T}W_{y_{i}}\right)}}$$ (2)

The maximum of *ρ*, which corresponds to the maximum canonical correlation between *X* and *Y*_*i*_, is taken as the recognition basis for stimulus frequency *f*_*i*_(*i* = 1, …, *K*).

When CCA is performed separately on each stimulus frequency *f*_*i*_(*i* = 1, …, *K*) and the respective maximum correlation coefficient $\rho_{f_{i}}$ is obtained, the target with stimulus frequency of *f*_*t*arg*et*_ can be judged by

$$f_{t\text{arg}et}=\underset{i=1,\ldots ,K}{\text{max}\rho_{f_{i}}}$$ (3)

In CCA calculations, the stimulus frequency *f*_*i*_(*i* = 1, …, 4) was selected as the stimulation frequency of each stimulator, the channel count of *C* was selected as 1, and the harmonics of *H* was selected as 0.5 and 1. Under the time window of 5 s per epoch, the offline recognition accuracy of each run was estimated as the percentage of correctly judged trials.

### 5 Statistical Analysis

To evaluate the mental load and fatigue level of the subjects, the changes of three EEG power indices *α*, *θ* and *θ* + *α*, and one ratio index *θ*/*α* [20–23], and two SSVEP and SSMVEP properties of amplitude and SNR in each run along the experiments were investigated. For offline analysis, signals from Oz channel were first band-pass filtered between 3 and 45 Hz with a 4th-order zero-phase-shift Butterworth filter. The power changes of the EEG signals in the *α* band of 8–13 Hz and the *θ* band of 4–7 Hz were calculated using Welch’s power spectral density estimation method in bins of 0.2 Hz. Then, the power indices *α* and *θ* and ratio index *θ/α* were used in mental load detection, and the power indices *α*, *θ* and *θ* + *α* were used at fatigue level detection. The mean values and SD of each index in the 1–5, 6–10, 11–15 and 16–20 epochs of each run in both SSVEP-BCI and SSMVEP-BCI tasks were used to represent the corresponding four different fatigue levels (i.e., level 1 level 2, level 3 and level 4), respectively. For the convenience of between subject comparisons and cross-subject analysis, it was proposed to normalize the three power indices *α*, *θ* and *θ* + *α*, and one ratio index *θ*/*α* for each subject by his/her respective maximal values among SSVEP-BCI and SSMVEP-BCI tasks [24].

The amplitudes and SNRs of SSVEP and SSMVEP responses at the stimulation frequency of 15 Hz were extracted by Fast Fourier Transform (FFT) in the respective epochs. The SNR was computed as the ratio of Fourier power at the target frequency to the mean value of its *n*-adjacent frequencies power:

$$SNR=\frac{n\times y(f)}{\Sigma_{k=1}^{n/2}\left[y(f+0.2\times k)+y(f-0.2\times k)\right]}$$ (4)

where *y* was the amplitude spectrum calculated by FFT, *f* was the stimulation frequency, and *n* = 6 was used so that the frequencies *f* ± 0.6 Hz were taken into account [25, 26].

One-way and two-way analysis of variance (ANOVA) with the criterion for statistical significance of *p* < 0.05 was employed to evaluate the statistics significance of changes in power and ratio indices for SSVEP-BCI and SSMVEP-BCI tasks.

## Results

### 1 Effects of Mental Load

The mental load analysis was restricted to unnormalized *α* and *θ* power. Flickering SSVEP and motion-reversal SSMVEP findings concerning mental load were carried out in both of between evoked EEG and ongoing EEG condition and within evoked EEG condition. It is hypothesized that mental load is associated with task difficulty and when performing BCIs the ongoing condition that the subjects did not attend any stimulator would have apparent lower mental load than visual attention condition that include a task, reflecting as a decrease in the ratio index *θ/α*. So, the explorative comparison of mental load between evoked EEG and ongoing EEG was performed first to validate the following mental load comparison between flickering SSVEP-BCI and motion-reversal SSMVEP-BCI tasks.

**Fig 1:**
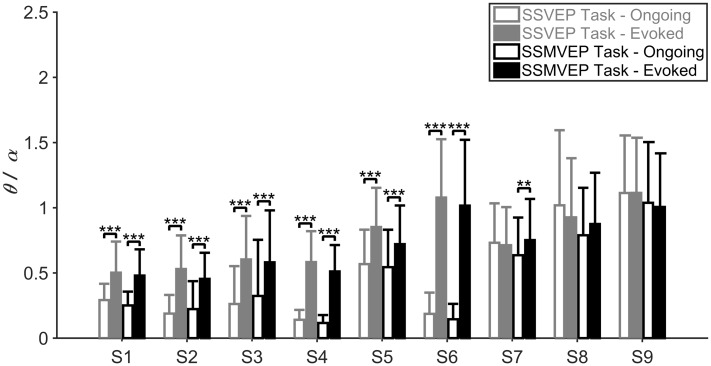
Comparison of mental load between the evoked EEG and ongoing EEG in both of flickering SSVEP-BCI and motion-reversal SSMVEP-BCI tasks for individual subjects. The mean values and SD of the ratio index *θ*/*α* of individual subjects within SSVEP-BCI and SSMVEP-BCI tasks were calculated. All statistics were assessed by unbalanced one-way ANOVA, *** *p* < 0.001 between evoked EEG and ongoing EEG, ** *p* < 0.01 between evoked EEG and ongoing EEG.

Fig 1 illustrated the ratio index *θ*/*α* calculated from evoked and ongoing EEG of flickering SSVEP-BCI and motion-reversal SSMVEP-BCI tasks. The data from the ISIs served as ongoing EEG since they did not include visual attention. Evoked and ongoing EEG indices were measured from the epoch and ISI periods where each run consisted of 20 epoch periods and 19 ISI periods, respectively. Unbalanced one-way ANOVA test of the ratio index *θ*/*α* revealed that the difference between evoked EEG and ongoing EEG was significant in both of flickering SSVEP BCI and motion-reversal SSMVEP BCI, and significantly lower *θ*/*α* values during the ongoing condition can be found as compared to the task condition for most subjects except Subject S8 and S9, where Subject S1 to Subject S6 (unbalanced one-way ANOVA: *p* < 0.0001 for all comparisons) showed significant difference in both of flickering SSVEP BCI and motion-reversal SSMVEP BCI. It implied that low theta and high alpha activities were associated with the low degree of mental load. Subject S7 also exhibited the similar results but only in the motion-reversal SSMVEP BCI (*F* = 7.12, *p* = 0.0083). Exceptionally, subject S8 and S9 presented inconspicuous mental load variation between evoked EEG and ongoing EEG condition, which may be related to the strong individual differences in the sensitivity of theta and alpha power on mental load [27, 28].

**Fig 2:**
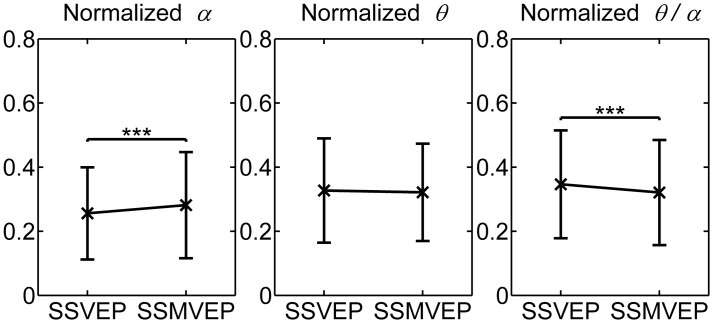
Comparison of the mean values and SD of mental load score between flickering SSVEP-BCI and motion-reversal SSMVEP-BCI task conditions over subjects. The mean values and SD of the normalized power indices *α* and *θ* and ratio index *θ*/*α* were calculated from evoked EEG over nine subjects. Horizontal axis: SSVEP—flickering SSVEP-BCI task condition; SSMVEP—motion-reversal SSMVEP-BCI task condition. All statistics were assessed by one-way ANOVA, *** *p* < 0.001 between evoked EEG in SSVEP-BCI and SSMVEP-BCI tasks.

Fig 2 showed the normalized power indices *α* and *θ* and corresponding ratio index *θ*/*α* that were calculated from evoked EEG of flickering SSVEP-BCI and motion-reversal SSMVEP-BCI tasks over nine subjects. Results were normalized per subject with respect to his/her maximal values among SSVEP-BCI and SSMVEP-BCI tasks. As hypothesized, a trend representing the alleviation of mental load as the decrease of the normalized *θ* power seemed to be present in SSMVEP-BCI task in comparison to SSVEP-BCI task over subjects, but was less significant (one-way ANOVA: *F* = 0.73, *p* = 0.3927). And a reversed effect of a significant increase in the normalized *α* power can be observed in SSMVEP-BCI task in comparison to SSVEP-BCI task (*F* = 15.57, *p* < 0.0001), which indicated a low degree of mental load occurred in SSMVEP BCI. Furthermore, difference in the normalized ratio index *θ/α* between the two mental conditions was also significant (*F* = 13.64, *p* = 0.0002).

### 2 Effects of Fatigue

**Fig 3:**
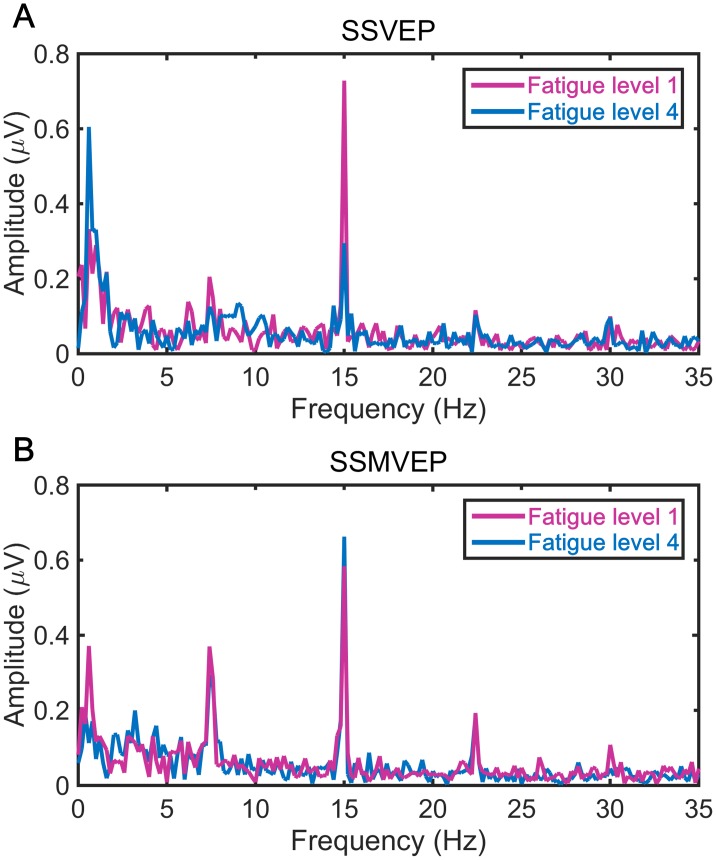
Grand-averaged SSVEP and SSMVEP spectra at fatigue level 1 and level 4 states over nine subjects. (A) Grand-averaged SSVEP spectrum. (B) Grand-averaged SSMVEP spectrum. Fatigue level 1: spectral power calculated from the first five epochs of each run in SSVEP-BCI and SSMVEP-BCI tasks; Fatigue level 4: spectral power calculated from the last five epochs of each run.

The spectral power of grand-averaged SSVEP and SSMVEP from the 1–5 and 16–20 epochs of each run, which represented fatigue level 1 and level 4 in both of flickering SSVEP-BCI and motion-reversal SSMVEP-BCI tasks, was depicted in Fig 3. Among the nine subjects, there was a trend for post-viewing reduction as compared to the initial viewing in both of the SSVEP and SSMVEP spectra at the stimulation frequency of 15 Hz. A detailed analysis indicated that the SSVEP spectra at fatigue level 4 were significantly smaller than its fatigue level 1 state as predicted (one-way ANOVA: *F* = 6.36, *p* = 0.0213, Fig 3A). But no significant fatigue effect was found within motion-reversal SSMVEP-BCI task (*F* = 0.1, *p* = 0.7522, Fig 3B). Corresponding CCA results also demonstrated significant offline accuracy decrease at fatigue level 4 state of SSVEP-BCI task (74.33% ± 10.83) as compared to its fatigue level 1 state (88.52% ± 11.07; one-way ANOVA: *F* = 7.56, *p* = 0.0143), whereas no significant difference (*F* = 0.12, *p* = 0.7351) can be found between fatigue level 1 accuracies (83.33% ± 15.90) and fatigue level 4 accuracies (85.56% ± 11.06) within SSMVEP-BCI task, and between fatigue level 1 states in both of SSVEP-BCI and SSMVEP-BCI tasks (*F* = 0.64, *p* = 0.4337). More specifically, the motion-reversal SSMVEP-BCI task at fatigue level 4 achieved higher recognition accuracies than flickering SSVEP-BCI task at fatigue level 4 (*F* = 4.73, *p* = 0.0449).

**Fig 4:**
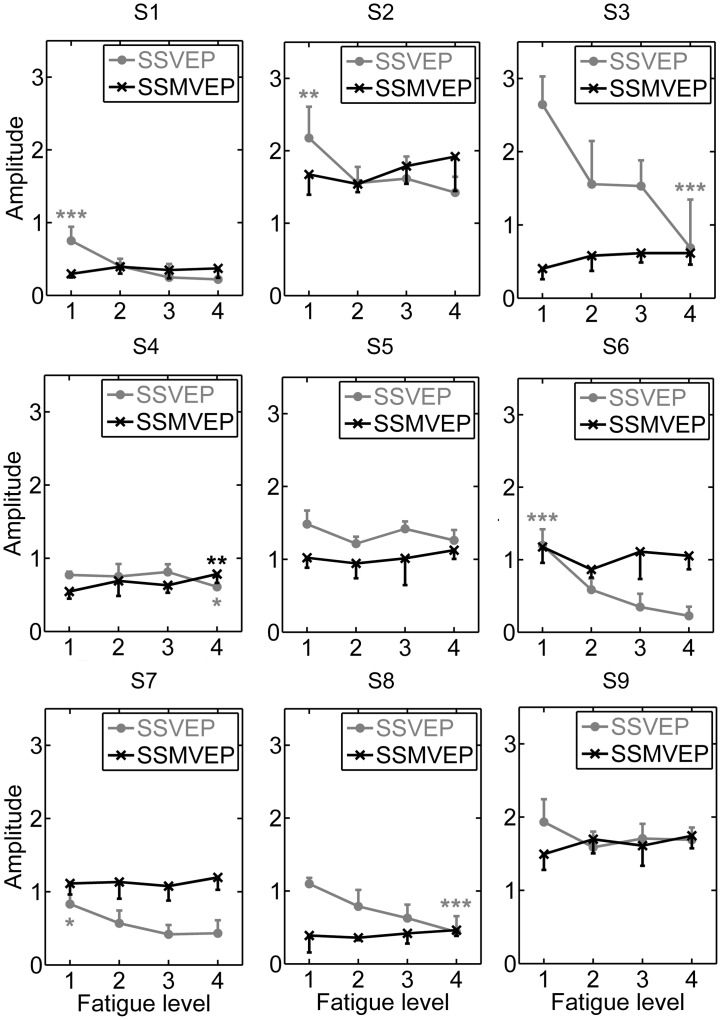
Comparison of SSVEP and SSMVEP amplitudes between four different fatigue levels for individual subjects. All statistics were assessed by one-way ANOVA, *** *p* < 0.001 between four different fatigue levels in both of SSVEP-BCI and SSMVEP-BCI tasks, ** *p* < 0.01 between four different fatigue levels, * *p* < 0.05 between four different fatigue levels.

**Fig 5:**
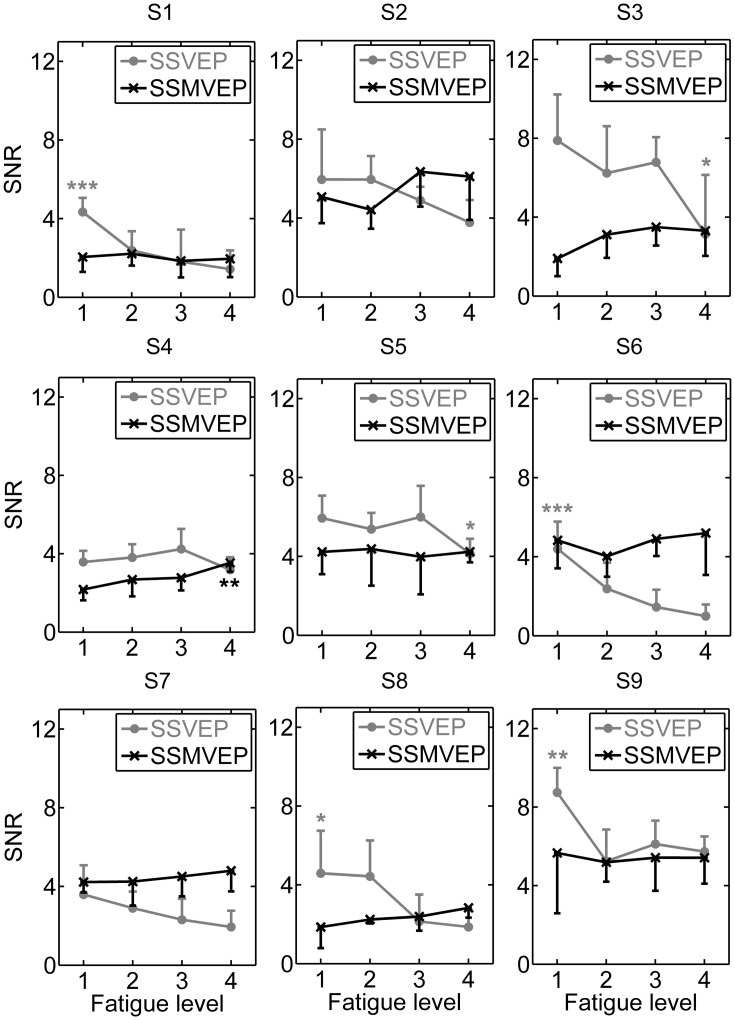
Comparison of SSVEP and SSMVEP SNRs between four different fatigue levels for individual subjects. All statistics were assessed by one-way ANOVA, *** *p* < 0.001 between four different fatigue levels in both of SSVEP-BCI and SSMVEP-BCI tasks, ** *p* < 0.01 between four different fatigue levels, * *p* < 0.05 between four different fatigue levels.

More sophisticated statistical analysis was illustrated in Figs 4 and 5. The amplitude and SNR differences between different fatigue levels for both of flickering and motion reversal tasks were analyzed in individual subjects, where amplitudes and SNRs at the stimulation frequency of 15 Hz were extracted from the spectral power of the averaged multi-run data of the same epoch order. The mean values and SD of the amplitudes and SNRs summed over the stimulation frequency of 15 Hz in the 1–5, 6–10, 11–15 and 16–20 epochs of multiple runs were grouped to represent fatigue level 1, 2, 3 and 4, respectively.

In Figs 4 and 5, we can see that the SSVEP trace behaved as a dramatically decrease in amplitudes and SNRs while the SSVEP traced behaved as a roughly stable or even increasing tendency as time elapsed. Subsequent two-way ANOVA test revealed that the interaction of the factors of “stimulation type” and “fatigue level” yielded significance in amplitudes and SNRs, especially for Subject S1, S2, S3, S4, S6, S7, S8 and S9 (*p* < 0.05 for amplitude and SNR comparisons). This implied that the factor of “stimulation type” have significant influence on the evolution of BCI performance during prolonged usage for most subjects. In Fig 4, one-way ANOVA revealed that the SSVEP amplitudes significantly decreased 71% (one-way ANOVA, post-hoc Tukey test, *F* = 27.01, *p* = 0.0008) from fatigue level 1 state to fatigue level 4 state in Subject 1, while the same trend of variation of 35% (*F* = 12.11, *p* = 0.0083), 74% (*F* = 32.77, *p* < 0.0004), 21% (*F* = 6.08, *p* = 0.0389), 81% (*F* = 72.27, *p* < 0.0001), 48% (*F* = 6.47, *p* = 0.0345) and 60% (*F* = 40.38, *p* = 0.0002) can be found in Subject S2, S3, S4, S6, S7 and S8, respectively. The same trend of decrease can also be found in Subject S5 and S9; however, this effect does not reach statistical significance (*p* > 0.05). On the other hand, the motion-reversal stimulation did not present significant amplitude decrement between different fatigue levels and even exhibited reversed phenomenon that most subjects presented higher responses as the fatigue level increased, especially for Subject S4 (one-way ANOVA, post-hoc Tukey test, *F* = 11.89, *p* = 0.0087). The similar SNR results can also be found in Fig 5.

Accompanying the above-mentioned CCA results that the motion-reversal SSMVEP-BCI task achieved comparable recognition accuracies with flickering SSVEP-BCI task at fatigue level 1, but much higher recognition accuracies than that of flickering SSVEP-BCI task at fatigue level 4, under the factor of “fatigue level”, the motion-reversal stimulation at fatigue level 1 achieved significantly smaller amplitudes and SNRs than the SSVEP responses in figure level 1, especially for Subject S1, S3, S4, S5, S8 and S9 (*p* < 0.05 for amplitude and SNR comparisons), but were comparable or even significantly larger at fatigue level 4 than the SSVEP responses in same fatigue level 4 for most subjects, especially for Subject S2, S6 and S7 (*p* < 0.05 for amplitude and SNR comparisons). This verified the significant interaction of factors of “stimulation type” and “fatigue level” that SSMVEP starts at lower amplitude and SNR, but does not decrease or even increase with time, whereas SSVEP starts higher, but then decreases dramatically as time elapsed. So it is demonstrated that motion-reversal SSMVEP stimulation exhibited a superior anti-fatigue efficacy and even better performance over conventional flickering SSVEP paradigm during prolonged BCI usage.

More specific mean values and SD of SSVEP and SSMVEP amplitude variations under fatigue level 1 and 4 for individual subjects were summarized in Table 1. As a supplement for the illustration in Fig 4, in Fig 5 the SNR results revealed an overall similar anti-fatigue effect in motion-reversal SSMVEP-BCI task, where the SNRs of SSMVEP at fatigue level 4 were much comparable or better than that at fatigue level 1. More specific mean values and SD of SSVEP and SSMVEP SNR variations under fatigue level 1 and 4 for individual subjects were listed in Table 2.

**Table 1:** Statistical summary of the amplitude variations under fatigue level 1 and 4 for individual subjects.

Subjects	SSVEP Level 1 Mean (SD)	SSVEP Level 4 Mean (SD)	ANOVA		SSMVEP Level 1 Mean (SD)	SSMVEP Level 4 Mean (SD)	ANOVA	
			*F*	*p*			*F*	*p*
**S1**	0.75 (0.19)	0.22 (0.12)	27.01	0.0008 ***	0.30 (0.05)	0.37 (0.13)	1.43	0.2665
**S2**	2.18 (0.43)	1.42 (0.22)	12.11	0.0083 **	1.67 (0.28)	1.92 (0.48)	1.00	0.3471
**S3**	2.64 (0.38)	0.69 (0.66)	32.77	0.0004 ***	0.40 (0.14)	0.62 (0.16)	4.94	0.0569
**S4**	0.77 (0.05)	0.61 (0.14)	6.08	0.0389 *	0.55 (0.10)	0.78 (0.12)	11.89	0.0087 **
**S5**	1.48 (0.19)	1.26 (0.14)	4.56	0.0653	1.02 (0.14)	1.13 (0.12)	1.60	0.2411
**S6**	1.20 (0.22)	0.23 (0.13)	72.27	< .0001 ***	1.18 (0.22)	1.05 (0.19)	0.91	0.3686
**S7**	0.83 (0.30)	0.43 (0.18)	6.47	0.0345 *	1.11 (0.15)	1.20 (0.17)	0.67	0.4374
**S8**	1.10 (0.08)	0.44 (0.22)	40.38	0.0002 ***	0.39 (0.23)	0.46 (0.08)	0.46	0.5185
**S9**	1.93 (0.31)	1.69 (0.17)	2.34	0.1646	1.49 (0.21)	1.75 (0.17)	4.21	0.0744

Level 1 Mean: mean values of the first five epochs in the multi-run data; Level 4 Mean: mean values of the last five epochs in the multi-run data; SD: standard deviation.

*** Significant at *p* = 0.001

** significant at *p* = 0.01

* significant at *p* = 0.05

**Table 2:** Statistical summary of the SNR variations under fatigue level 1 and 4 for individual subjects.

Subjects	SSVEP Level 1 Mean (SD)	SSVEP Level 4 Mean (SD)	ANOVA		SSMVEP Level 1 Mean (SD)	SSMVEP Level 4 Mean (SD)	ANOVA	
			*F*	*p*			*F*	*p*
**S1**	4.34 (0.72)	1.43 (0.95)	29.43	0.0006 ***	2.05 (0.76)	1.96 (0.93)	0.03	0.8675
**S2**	5.96 (2.54)	3.78 (1.14)	3.07	0.1177	5.07 (1.33)	6.11 (2.20)	0.81	0.3939
**S3**	7.89 (2.33)	3.14 (3.00)	7.79	0.0235 *	1.91 (0.90)	3.31 (1.27)	4.08	0.0779
**S4**	3.58 (0.57)	3.18 (0.65)	1.08	0.3291	2.17 (0.55)	3.54 (0.45)	18.18	0.002 **
**S5**	5.94 (1.14)	4.14 (0.76)	8.64	0.0187 *	4.23 (1.13)	4.24 (0.54)	< .01	0.9883
**S6**	4.40 (1.38)	0.99 (0.59)	25.70	0.0010 ***	4.83 (1.42)	5.20 (2.12)	0.10	0.7561
**S7**	3.59 (1.48)	1.94 (0.83)	4.72	0.0615	4.23 (0.53)	4.80 (1.05)	1.21	0.3038
**S8**	4.59 (2.16)	1.87 (0.87)	6.83	0.0309 *	1.86 (1.07)	2.83 (0.50)	3.41	0.1022
**S9**	8.75 (1.25)	5.73 (0.77)	20.88	0.0018 **	5.67 (3.07)	5.42 (1.32)	0.03	0.8728

Level 1 Mean: mean values of the first five epochs in the multi-run data; Level 4 Mean: mean values of the last five epochs in the multi-run data; SD: standard deviation.

*** Significant at *p* = 0.001

** Significant at *p* = 0.01

* significant at *p* = 0.05

**Fig 6:**
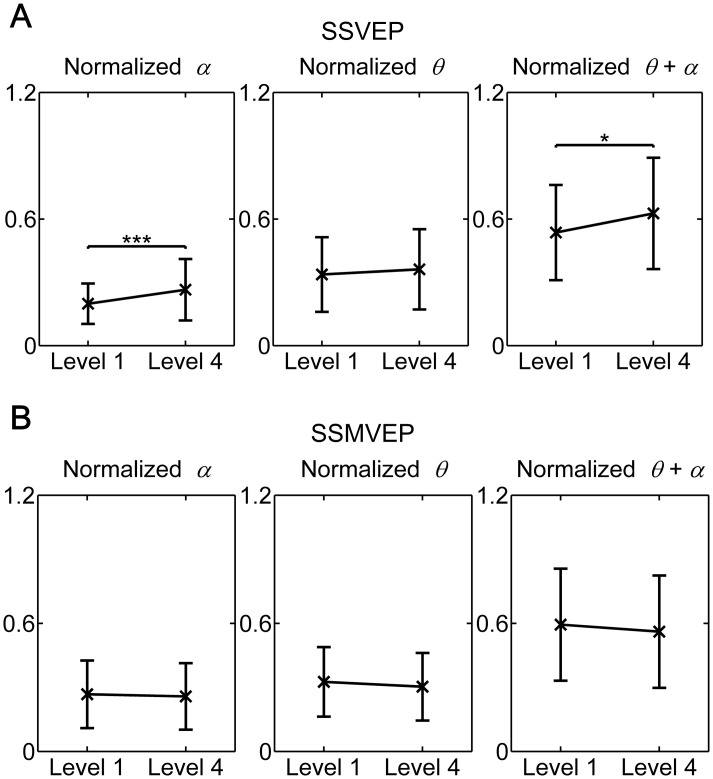
Comparison of the mean values and SD of fatigue score between fatigue level 1 and 4 in both of flickering SSVEP-BCI and motion-reversal SSMVEP-BCI conditions over subjects. (A) Comparison of fatigue between fatigue level 1 and 4 in SSVEP-BCI condition. (B) Comparison of fatigue between fatigue level 1 and 4 in SSMVEP-BCI condition. The mean values and SD of the normalized power indices *α*, *θ* and *θ* + *α* were calculated from ongoing EEG over nine subjects. Horizontal axis: Level 1—fatigue level 1 state; Level 4—fatigue level 4 state. All statistics were assessed by one-way ANOVA, *** *p* < 0.001 between ongoing EEG in SSVEP-BCI and SSMVEP-BCI conditions, * *p* < 0.05 between ongoing EEG in SSVEP-BCI and SSMVEP-BCI conditions.

Fig 6 presented the normalized power indices *α*, *θ* and *θ* + *α* for each of flickering SSVEP-BCI and motion-reversal SSMVEP-BCI conditions over nine subjects. The ongoing EEG has been proven to be closely related to changes in the level of fatigue in previous studies [13,29]. In the present study, the spectral powers of *α*, *θ* and *θ* + *α* over the first and last five ISIs of all experimental runs were extract to represent fatigue level 1 and 4, respectively. Indices were normalized per subject with respect to his/her maximal values among SSVEP-BCI or SSMVEP-BCI conditions.

Over all subjects, a general increase tendency in the normalized *α*, *θ* and *θ + α* indices has been observed in both of SSVEP-BCI and SSMVEP-BCI tasks as a consequence of the prolonged BCI usage. One-way ANOVA revealed a decreased tendency but no significant difference in the normalized *α*, *θ* and *θ + α* indices between initial-viewing and post-viewing conditions (*F* = 0.25, *p* = 00.6207 for *α* index, *F* = 1.19, *p* = 0.276 for *θ* index and *F* = 0.15, *p* = 0.698 for *θ + α* index) within SSMVEP-BCI task. But an expected high mental fatigue with a statistically significant increase of power in the *α* band occurred within the flickering SSVEP-BCI task (*F* = 17.37, *p* < 0.0001), and an effect in the *θ* index seemed to be present, but was less clear (*F* = 1.05, *p* = 0.3056). Subsequent power index *θ + α* was also significant (*F* = 6.16, *p* = 0.0138), which was in accordance with the EEG study by Macchi et al. [30] that *θ* waves alone showed insignificant effect, but the index containing both *θ* and *α* showed significant effect.

## Discussion

The theta activity occurs in a variety of mental states associating with drowsiness, or starting to sleep, attention, and processing of cognitive and perceptual information. Evidence for an association between theta and mental effort has been summarized in several reviews [31–33] and the increase in theta activity is related to performance decrements on task. The alpha waves appear during relaxed condition, at decreased attention levels and in a drowsy but wakeful state, and the increased alpha power associating with fatigue is related to the increased mental effort to maintain vigilance level. Previous studies [34, 35] showed that the band power in the theta frequency bands increases with mental load, while the band power in the alpha band decreases. Regarding fatigue, or reduced alertness, it induces an increase of band power in the theta and alpha frequency [33].

This study investigated on the mental load and fatigue effects with the design factors of periodic flickering and motion-reversal patterns. Results showed that conventional periodic flickering paradigm achieved a significant increase in *θ* power and in the ratio index *θ*/*α* during the visual attention task as compared to motion-reversal paradigm, indicating a higher mental load requirements when performing the task. Considering the previous studies concerning the relationship between mental load and EEG responses that high mental load may result in reduced EEG amplitudes [1, 13], the severely reduced SSVEP amplitudes and SNRs after prolonged viewing reflected a decrease in subjects’ attentional level, which may be induced by the high mental load in the operation of SSVEP-BCI task that increases in task difficulty and mental load may lead to drowsiness and the increased mental effort to stay wakeful and cause fatigue. The increased *α* and *θ* + *α* band power also illustrated a more severe fatigue level in the post-viewing condition, which was consistent with previous researches on fatigue monitoring. Furthermore, the interaction between the factors of “stimulation type” and “fatigue level” yielded significance in amplitudes and SNRs, implying the factor of “stimulation type” have significant influence on the evolution of BCI performance during prolonged usage, where the tasks exposure at motion-reversal stimulation did not presented significant amplitude and SNR variations throughout the 195-s experimental time course. The even higher elevation of offline recognition accuracies, amplitudes and SNRs than SSVEP-BCI implementation were also presented in the post-viewing condition in most subjects, revealing the interaction effect that the stimulation type of motion reversal would maintain sustained performance throughout actual BCI implementations, whereas the stimulation type of flashing suffers from short-term fatigue and is suitable for short-time usage (e.g., within 30 min) due to its excellent initial performance. Besides, there were also no significant power indices changes in the evoked and ongoing EEG responses in SSMVEP condition, making the periodic motion-reversal pattern a user-friendly design to alleviate the users’ mental load and fatigue effects with minimal influence on steady-state EEG response properties in favor of actual long-term usage.

According to classical theory, the visual system was classified into two major pathways of the parvocellular (PC) pathway and the magnocellular (MC) pathway. The MC pathway is involved in the detection of dynamic motion and depth, whereas the PC pathway is involved in the detection of spatial contrasts and color information, with a slower propagation than the MC pathway. Russo and Spinelli [36] concluded that spatial attention uses different mechanism to affect sensory transmission in the MC and PC systems. It was proposed that attention uses the faster and more dominant signals of the MC pathway to give priority to stimuli at attended locations and simultaneously enhance activity of the PC pathway. A weakened or slowed MC projection may inhibit visual attention, and if attentional networks are more reliant on PC pathways, extra reaction time is requires for attention [37], which would lead to the increase of attention demand [38]. So the attention demand may be alleviated due to the motion-reversal design, thus the long-term mental load and fatigue outcome was reduced substantially by scaling down the demands on the visual system, and on the attention to the motion-reversal SSMVEP-BCI task.

To conclude, this study has quantitatively explored the effect of alleviating mental load and corresponding anti-fatigue performance in the periodic motion-reversal task as compared to conventional flickering based visual attention task, which verified the feasibility and effectiveness of the proposed measures and provided an objective method guiding the design of more practically applicable BCI systems with alleviated mental load and anti-fatigue solution for long-term operation. The involvement of more stimulation frequencies as well as other potential influences would be addressed in future studies.

## Supporting Information

S1 File The *θ*/*α* ratio index. (MAT)

S2 File The power of *θ* and *α* bands. (MAT)

S3 File The averaged raw data across epochs and runs. (MAT)

S4 File The averaged power spectrum and SNR across runs. (MAT)

S1 Table Offline CCA discrimination results. (DOC)
