# Supplementary material for: Effects of Mental Load and Fatigue on Steady-State Evoked Potential Based Brain Computer Interface Tasks: A Comparison of Periodic Flickering and Motion-Reversal Based Visual Attention
Source: PLoS One. 2016 Sep 22;11(9):e0163426. doi: 10.1371/journal.pone.0163426 (PMC5033480; doi:10.1371/journal.pone.0163426)
Supplement: S1 Table — (DOC) [file pone.0163426.s005.DOC]

**S1 Table. Offline CCA discrimination results.**

|  | **SSVEP CCA Accuracy (%)** | | **SSMVEP CCA Accuracy (%)** | |
| --- | --- | --- | --- | --- |
| **Subjects** | **First 5 Epochs** | **Last 5 Epochs** | **First 5 Epochs** | **Last 5 Epochs** |
| **S1** | 80 | 70 | 100 | 100 |
| **S2** | 93.33 | 83.33 | 83.33 | 90 |
| **S3** | 100 | 67.5 | 80 | 80 |
| **S4** | 66.67 | 60 | 50 | 63.33 |
| **S5** | 100 | 85 | 100 | 80 |
| **S6** | 90 | 73.33 | 76.67 | 96.67 |
| **S7** | 80 | 64 | 80 | 90 |
| **S8** | 90 | 72.5 | 100 | 80 |
| **S9** | 96.67 | 93.33 | 80 | 90 |
